# GABAergic Regulation of Astroglial Gliotransmission through Cx43 Hemichannels

**DOI:** 10.3390/ijms232113625

**Published:** 2022-11-07

**Authors:** Ivanka Jiménez-Dinamarca, Rachel Reyes-Lizana, Yordan Lemunao-Inostroza, Kevin Cárdenas, Raimundo Castro-Lazo, Francisca Peña, Claudia M. Lucero, Juan Prieto-Villalobos, Mauricio Antonio Retamal, Juan Andrés Orellana, Jimmy Stehberg

**Affiliations:** 1 Laboratorio de Neurobiología, Instituto de Ciencias Biomédicas, Facultad de Medicina, Universidad Andres Bello, Santiago 8370186, Chile; 2 Centro de Fisiología Celular e Integrativa, Facultad de Medicina, Universidad del Desarrollo–Clínica Alemana, Santiago 7780272, Chile; 3 Departamento de Neurología, Escuela de Medicina and Centro Interdisciplinario de Neurociencias, Facultad de Medicina, Pontificia Universidad Católica de Chile, Santiago 8330024, Chile

**Keywords:** GABA, GABA_A_ receptors, astrocytes, Cx43 hemichannels, astroglia, connexin 43, gliotransmission, astroglial release

## Abstract

Gamma-Aminobutyric acid (GABA) is the primary inhibitory neurotransmitter in the brain. It is produced by interneurons and recycled by astrocytes. In neurons, GABA activates the influx of Cl^-^ via the GABA_A_ receptor or efflux or K^+^ via the GABA_B_ receptor, inducing hyperpolarization and synaptic inhibition. In astrocytes, the activation of both GABA_A_ and GABA_B_ receptors induces an increase in intracellular Ca^2+^ and the release of glutamate and ATP. Connexin 43 (Cx43) hemichannels are among the main Ca^2+^-dependent cellular mechanisms for the astroglial release of glutamate and ATP. However, no study has evaluated the effect of GABA on astroglial Cx43 hemichannel activity and Cx43 hemichannel-mediated gliotransmission. Here we assessed the effects of GABA on Cx43 hemichannel activity in DI NCT1 rat astrocytes and hippocampal brain slices. We found that GABA induces a Ca^2+^-dependent increase in Cx43 hemichannel activity in astrocytes mediated by the GABA_A_ receptor, as it was blunted by the GABA_A_ receptor antagonist bicuculline but unaffected by GABA_B_ receptor antagonist CGP55845. Moreover, GABA induced the Cx43 hemichannel-dependent release of glutamate and ATP, which was also prevented by bicuculline, but unaffected by CGP. Gliotransmission in response to GABA was also unaffected by pannexin 1 channel blockade. These results are discussed in terms of the possible role of astroglial Cx43 hemichannel-mediated glutamate and ATP release in regulating the excitatory/inhibitory balance in the brain and their possible contribution to psychiatric disorders.

## 1. Introduction

Gamma-Aminobutyric acid (GABA) is the primary inhibitory neurotransmitter in the adult brain, having a critical function in maintaining its inhibitory/excitatory balance, producing brain oscillations, and allowing several cognitive processes, including memory [1,2]. The dysfunction of GABAergic activity has been linked to many psychiatric disorders, including anxiety-associated disorders [3] and depression [4], and its agonism is the target of the most common anxiolytics, benzodiazepines.

GABA is released by inhibitory neurons known as interneurons. It acts by activating post-synaptic receptors belonging to two types; A and B. Type A receptors are ionotropic receptors that allow Cl^−^ influx, inducing a membrane hyperpolarization in neurons. Type B receptors are metabotropic receptors coupled to G-proteins linked to potassium channels, whose activation in neurons also induces hyperpolarization, but through the efflux of K^+^. Although GABA is mainly produced and released by interneurons, it is recycled from the synaptic cleft by astrocytes through transporters [5], and current evidence suggests that it can also be released by astrocytes into synapses as a gliotransmitter [6,7,8,9].

Astrocytes express both GABA_A_ [10,11,12] and GABA_B_ receptors [8,13,14]. Unlike neurons, GABA induces astroglial activation by increasing intracellular Ca^2+^ concentration [Ca^2+^]_i_ [15,16,17,18,19]. Studies have shown that the activation of GABA_A_ receptors leads to Cl^-^ efflux (instead of influx) from astrocytes, inducing depolarizing currents and triggering astrocytic Ca^2+^ signaling via the activation of voltage-sensitive Ca^2+^ channels [11,20,21,22,23]. The activation of GABA_B_ receptors in astrocytes has been reported to induce increases in [Ca^2+^]_i_ involving Gi/o proteins and Ca^2+^ release from intracellular stores via IP_3_ signaling [19,24].

Previous studies have shown that GABA triggers the [Ca^2+^]_i_-dependent release of glutamate [25,26] and ATP/adenosine [25,27,28] from astrocytes. Even though both glutamate and ATP are excitatory, the net result of astroglial glutamate and ATP release from astrocytes after GABA exposure is inhibitory. The latter occurs via the neuronal conversion of ATP into adenosine, and concomitant activation of neuronal adenosine receptors, which appears to be critical for the modulation of inhibitory interneuron activity [25,27,28].

The [Ca^2+^]_i_-dependent release of gliotransmitters in astrocytes takes place via different cellular mechanisms, among which, connexin 43 (Cx43) hemichannels appear to be important for the release of both glutamate and ATP [29]. No study to date has evaluated the effect of GABA on astroglial Cx43 hemichannel activity and Cx43 hemichannel-mediated gliotransmission. Here we evaluated the effects of GABA on Cx43 hemichannel activity and gliotransmission in DI TNC1 rat astrocytes, by measuring DAPI uptake, and in rat hippocampal slices, by measuring Etd^+^ uptake, as well as extracellular glutamate and ATP content, after incubation with GABA and the GABA_A_ and GABA_B_ receptor antagonists bicuculline and CGP55845, respectively.

## 2. Results

Astroglial Cx43 hemichannels open in response to increments in [Ca^2+^]_I_ or when the incubating medium lacks Ca^2+^ [30]. When incubating cells in a medium lacking Ca^2+^, to avoid potential interference from extracellular Mg^2+^, which can decrease Cx43 hemichannel opening [31], the solution is made free of divalent cations and includes EGTA as an extracellular Ca^2+^ chelator (Ca^2+^-free condition) [32]. In such condition, Cx43 hemichannels have a high opening probability, and the capacity of neurotransmitters to induce their closure can be assessed. In contrast, when incubating astrocytes in normal [Ca^2+^] (~1–2 mM), the Cx43 hemichannel opening probability is low, so neurotransmitters can be evaluated for their capacity to increase hemichannel activity.

**Figure 1:**
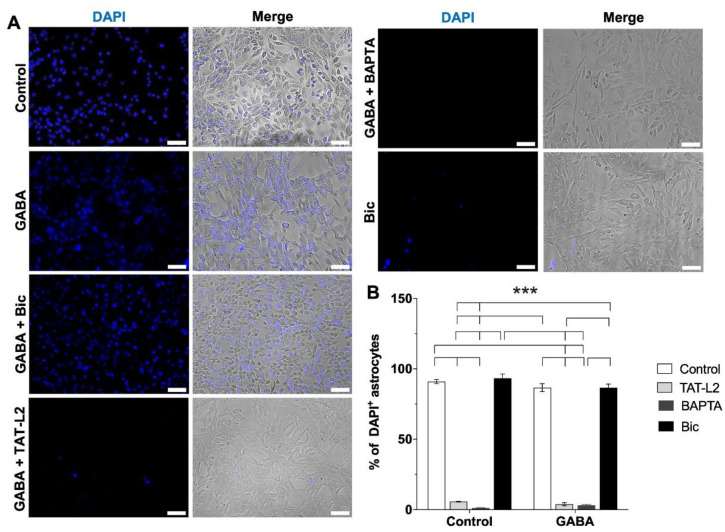
Increased DAPI uptake in DI TNC1 astrocytes incubated in a Ca^2+^-free solution is mediated by astroglial Cx43 hemichannels and depends on intracellular [Ca^2+^] but is unaffected by GABA or GABA_A_ receptor antagonist Bicuculine. (**A**) Representative photomicrographs showing increased DAPI uptake in DI TNC1 astrocytes incubated in a Ca^2+^-free culture medium. Increased DAPI uptake was blunted by 10 nM TAT-Cx43L2 (TAT-L2) or 100 nM BAPTA-AM (BAPTA), but unaffected by 1 µM GABA alone (GABA) or 100 µM bicuculline (Bic). Scale bar: 50 µm. (**B**) Quantification of DAPI uptake. *n =* 3, with technical triplicates. Error bars: SE; *** *p* ˂ 0.001.

Under the extracellular Ca^2+^-free condition, as expected, a significant increase in DAPI uptake can be observed in DI TNC1 rat astrocytes (Figure 1A,B, control), effect that was blocked by the selective Cx43 hemichannel blocker peptide TAT-Cx43L2 (Figure 1A,B, TAT-L2) and the cell-permeable Ca^2+^ chelator BAPTA-AM (Figure 1A,B, BAPTA). The astroglial response to the lack of extracellular Ca^2+^ is known to be dependent on the release of Ca^2+^ from intracellular stores [33]. As the extracellular Ca^2+^-free solution already has a non-cell permeable Ca^2+^ chelator (EGTA), the effects of the cell-permeable BAPTA-AM are considered an effect of chelating intracellular Ca^2+^. Hence, the cell-permeable Ca^2+^ chelator BAPTA-AM prevents the increase in [Ca^2+^]_i_ induced by the lack of extracellular Ca^2+^, which is needed to trigger Cx43 hemichannel opening. Thus, our results confirm that astroglial DAPI uptake under extracellular Ca^2+^-free conditions is dependent on Cx43 hemichannels and [Ca^2+^]_i_.

Incubation with 1 μM GABA did not affect the increase in DAPI uptake in astrocytes incubated in the extracellular Ca^2+^-free solution (Figure 1A,B, GABA), which was also unaffected by GABA_A_ receptor antagonist bicuculline (Figure 1A,B, Bic). The above suggests that GABA does not induce the closure of Cx43 hemichannels.

**Figure 2:**
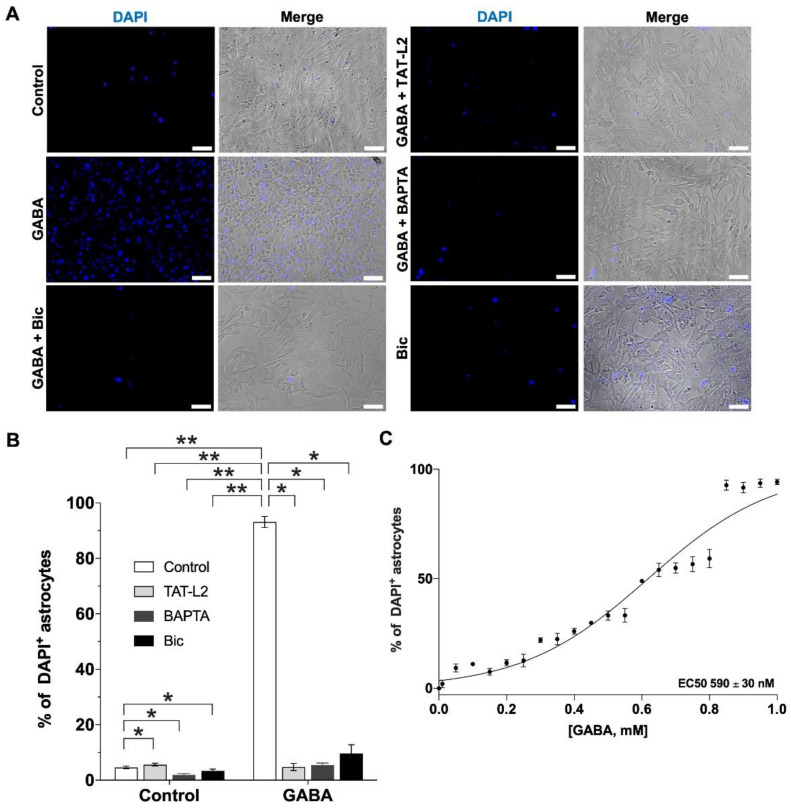
GABA increases DAPI uptake in DI TNC1 astrocytes through Cx43 hemichannels via activation of GABA_A_ receptors. (**A**) Representative photomicrographs showing DAPI uptake in DI TNC1 astrocytes under normal extracellular Ca^2+^ conditions (Control), show increased DAPI uptake in response to 1 µM GABA, effect that is prevented by preincubation with 100 µM bicuculline (Bic), 10 nM TAT-Cx43L2 (TAT-L2) or 100 nM BAPTA-AM (BAPTA). Scale bar: 50 µm. (**B**) Quantification of DAPI uptake. *n =* 3, with technical triplicates. (**C**) Concentration response curve for GABA on astroglial DAPI uptake performed to estimate the EC50 for GABA (EC50: 590 ± 30 nM). Error bars: SE; * *p* ˂ 0.05, ** *p* ˂ 0.01.

When DI TNC1 astrocytes were incubated in a solution with normal Ca^2+^ (1 mM), as expected, DAPI uptake was very low (Figure 2A,B, control). However, 1 μM GABA induced a large and significant increase in DAPI uptake (Figure 2A,B, GABA). The increased uptake in response to GABA was prevented by preincubation with the selective Cx43 hemichannel blocker TAT-Cx43L2 (Figure 2A,B, GABA + TAT-L2), suggesting that GABA induced an increase in Cx43 hemichannel activity. The effect of GABA on Cx43 hemichannel activity was [Ca^2+^]_i_-dependent, as it was prevented by the cell-permeable Ca^2+^ chelator BAPTA-AM (Figure 2A,B, GABA + BAPTA). To determine whether the effect of GABA was mediated by GABA_A_ or GABA_B_ receptors, cells were preincubated with either GABA_A_ receptor antagonist bicuculline or GABA_B_ receptor antagonist CGP55845 (CGP) before incubation with GABA. Bicuculline prevented the increase in DAPI uptake induced by GABA (Figure 2A, GABA + Bic). Bicuculline at the concentration used to block the effects of GABA did not affect Cx43 hemichannel activity by itself (Figure 2A,B, control + Bic). GABA showed an EC50 of 590 ± 30 nM (Figure 2C), and in total, 86.5% of the cells showed uptake after exposure to GABA, in stark contrast to the only 4.6% of astrocytes that showed basal DAPI uptake in control conditions (without GABA). In terms of fluorescence intensity, astrocytes showed similar median intensities between the control and GABA-treated conditions, showing only significant differences in the number of labeled cells (see Supplementary Figure S1A–C).

**Figure 3:**
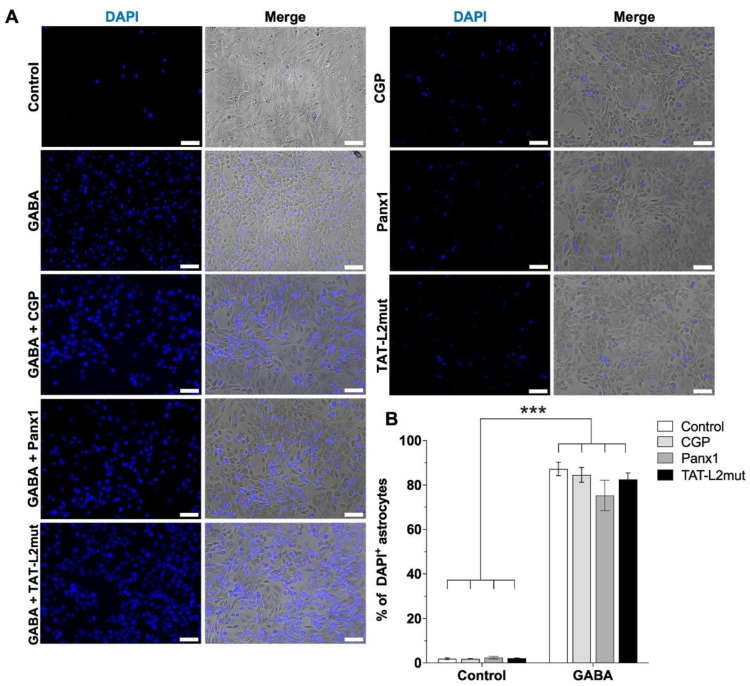
The increase in DAPI uptake in DI TNC1 astrocytes in response to GABA is not mediated by GABA_B_ receptors or Panx1 channels. (**A**) Representative photomicrographs showing DAPI uptake in DI TNC1 astrocytes under normal extracellular Ca^2+^ conditions (10 µM, control), showing increased DAPI uptake in response to 1 µM GABA, an effect that was unaffected by preincubation with 1 µM GABA_B_ receptor antagonist CGP55845 (CGP), 10 nM mutant TAT-Cx43L2 ^H126K/I130N^ (TAT-L2mut) or 150 nM ^10^Panx1 (Panx1). Scale bar: 50 µm. (**B**) Quantification of DAPI uptake. *n =* 3, with technical triplicates. Error bars: SE; *** *p* ˂ 0.001.

Although the effect of GABA was completely prevented by GABA_A_ receptor antagonist bicuculline, cells were also preincubated with GABA_B_ receptor antagonist CGP55845 (CGP, 1 µM), to assess a possible contribution of GABA_B_ receptors. As can be observed in Figure 3A,B, CGP), GABA_B_ receptor blockade by 1 µM CGP55845 had no effect on the GABA-induced increase in DAPI uptake.

Pannexin channels have also been associated with gliotransmitter release (reviewed in [29]). Although GABA-induced increase in DAPI uptake was completely prevented by Cx43 hemichannel blocker TAT-Cx43L2, we decided to evaluate the possible contribution of pannexin 1 (Panx1) channels. Hence, astrocytes were preincubated with Panx1 channel blocker peptide ^10^Panx1 (Panx1, 150 µM). DAPI uptake in response to GABA was unaffected by pannexin channel blockade (Figure 3, Panx1).

The above results suggest that the effect of GABA on DI TNC1 astroglial uptake is mediated by GABA_A_ receptors and by the activation of Cx43 hemichannels.

To further corroborate the specificity of the contribution of Cx43 hemichannels, DI TNC1 astrocytes were also preincubated with the mutant TAT-Cx43L2^H126K/I130N^ peptide (TAT-L2 mut), which is a peptide that differs from TAT-Cx43L2 by 2 amino acids, losing its affinity for Cx43, lacking inhibitory properties on Cx43-hemichannels [34], and used to assess the specificity of TAT-Cx43L2 on Cx43 hemichannel activity [35,36]. The mutated peptide did not affect GABA-induced increases in Cx43 hemichannel activity (Figure 3, TAT-L2mut), corroborating the specificity of the effects of the TAT-Cx43L2 peptide.

**Figure 4:**
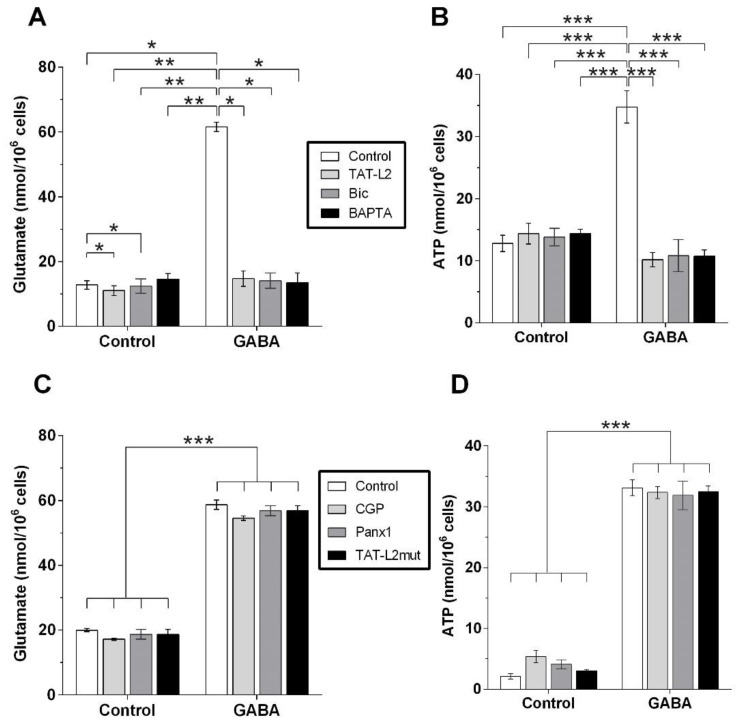
GABA induces the release of glutamate and ATP from DI TNC1 astrocytes, mediated by astroglial Cx43 hemichannels and the activation of GABA_A_ receptors. (**A**) The extracellular glutamate concentration was determined after incubation with 1 µM GABA alone (GABA) or after preincubation with 100 µM bicuculline (Bic), 10 nM TAT-Cx43L2 (TAT-L2) or 100 nM BAPTA-AM (BAPTA). (**B**) Extracellular ATP concentration was determined after incubation with 1 µM GABA alone or preincubated with 100 µM bicuculline (Bic), 10 nM TAT-Cx43L2 (TAT-L2) or 100 nM BAPTA-AM (BAPTA). (**C**) Extracellular glutamate concentration was determined after incubation with 1 µM GABA alone or preincubated with 1 µM CGP55845 (CGP), 10 nM mutant TAT-Cx43L2 (TAT-L2mut) or 150 nM ^10^Panx1 (Panx1). (**D**) Extracellular ATP concentration was determined after incubation with 1 µM GABA alone or preincubated with 1 µM CGP55845 (CGP), 10 nM mutant TAT-Cx43L2 (TAT-L2mut) or ^10^Panx1 (Panx1). *n* = 3, with technical triplicates. Error bars: SE; * *p* ˂ 0.05, ** *p* ˂ 0.01, *** *p* ˂ 0.001.

Analysis of the glutamate content in the culture medium in which DI TNC1 astrocytes were incubated with the different ligands showed that GABA induced the release of glutamate (Figure 4A, GABA), which was prevented by preincubation with TAT-Cx43L2 (Figure 4A, GABA + TAT-L2), BAPTA-AM (Figure 4A, GABA + Bapta) and GABA_A_ receptor antagonist bicuculline (Figure 4A, GABA + Bic). The latter suggesting that GABA induces the Cx43 hemichannel-dependent release of glutamate, which is [Ca^2+^]_i_-dependent and mediated by GABA_A_ receptors.

GABA also induced the release of ATP (Figure 4B, GABA), which was prevented by preincubation with TAT-Cx43L2 (Figure 4B, GABA + TAT-L2), BAPTA-AM (Figure 4B, GABA + Bapta), and GABA_A_ receptor antagonist bicuculline (Figure 4B, GABA + Bic), suggesting that GABA induces Cx43 hemichannel-dependent release of ATP, which is [Ca^2+^]_i_ -dependent and mediated by GABA_A_ receptors.

The GABA_B_ receptor antagonist CGP55845 had no effect on the astroglial release of glutamate in response to GABA (Figure 4C, CGP), suggesting that the GABA-induced release of glutamate was not mediated by the GABA_B_ receptor. Congruently, CGP55845 also had no effect on the astroglial release of ATP induced by GABA (Figure 4D, CGP). The above results suggest that DI TNC1 astrocytes release gliotransmitters glutamate and ATP in response to GABA, via the activation of GABA_A_ and not GABA_B_ receptors.

As with DAPI uptake, the release of glutamate and ATP in response to GABA was also not affected by preincubation with the Panx1 channel blocker peptide ^10^Panx1 (Figure 4C,D, Panx1). Likewise, preincubation with the mutant TAT-Cx43^L2H126K/I130N^ peptide also did not affect the release of glutamate or ATP in response to GABA (Figure 4C,D, TAT-L2 mut). This suggests that gliotransmission in response to GABA was mediated by Cx43 hemichannels, without a detectable contribution from Panx1 channels.

All the previous results were obtained from DI TNC1 astrocytes, which, arguably, being a cell line, may not completely recapitulate astroglia. Consequently, Cx43 hemichannel activity was measured using Etd^+^ in rat hippocampal slices in response to GABA, and the incubating medium was used to measure glutamate and ATP.

**Figure 5:**
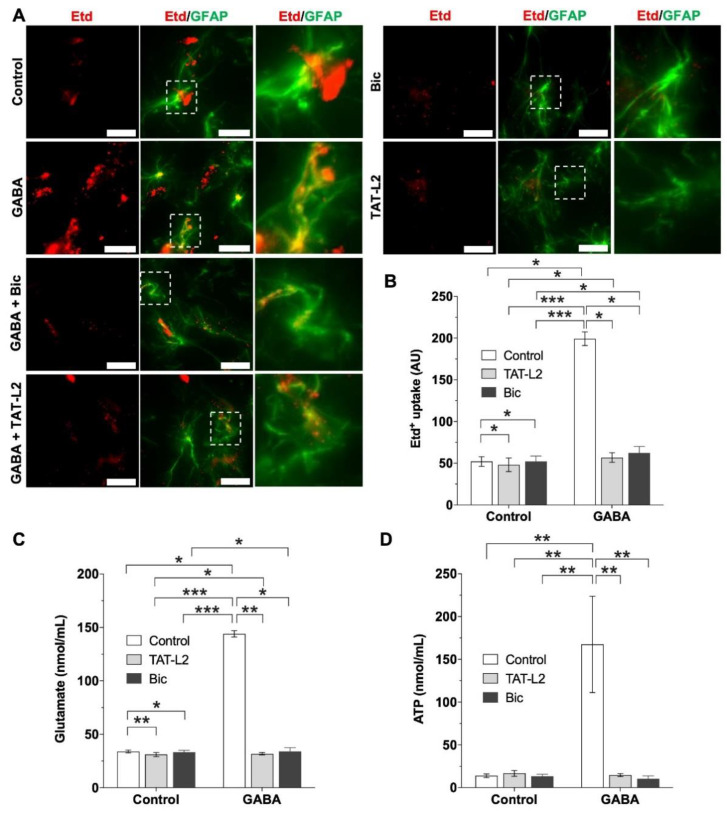
GABA increases astroglial Cx43 hemichannel activity in rat hippocampal slices via activation of GABA_A_ receptors. (**A**) Representative photomicrographs showing ethidium (Etd^+^) uptake (red) and GFAP immunolabeling (green) in hippocampal slices. Scale bar: 50 µm. (**B**) Quantification of Etd^+^ uptake. Increased Etd^+^ uptake was found in response to 1 µM GABA (GABA), an effect that was prevented by preincubation with 100 µM bicuculline (Bic) or 10 nM TAT-Cx43L2 (TAT-L2). (**C**) Extracellular glutamate concentration from the culture medium of hippocampal slices without ligands (control), or after incubation with 1 µM GABA alone (GABA), or after preincubation with 100 µM bicuculline (Bic) or 10 nM TAT-Cx43L2 (TAT-L2). (**D**) Extracellular ATP concentration without ligand (control) or after incubation with 1 µM GABA alone (GABA) or preincubated with 100 µM bicuculline (Bic) or 10 nM TAT-Cx43L2 (TAT-L2). In all cases, *n =* 3, with technical triplicates. Error bars: SE; * *p* ˂ 0.05, ** *p* ˂ 0.01 and *** *p* ˂ 0.001.

As can be seen in Figure 5A,B, GABA induced a large and significant increase in Etd^+^ uptake compared to the control condition (without GABA), an effect that was entirely blunted by preincubation with either TAT-Cx43L2 (TAT-L2) or bicuculline (Bic). The above results corroborate what was seen in DI TNC1 astrocytes and demonstrate that GABA induces an increase in Cx43 hemichannel activity in astrocytes from hippocampal slices, via activation of GABA_A_ receptors.

Analysis of the culture medium of the hippocampal slices after GABA incubation shows a large and significant increase in extracellular glutamate (Figure 5C, GABA), which was completely prevented by preincubation with bicuculline (Figure 5C, Bic) and with TAT-Cx43L2 (Figure 5C, TAT-L2). GABA also induced an increase in extracellular ATP (Figure 5D, GABA), which was averted by preincubation with bicuculline (Figure 5D, Bic) and with TAT-Cx43L2 (Figure 5D, TAT-L2). For detailed results see Supplementary Table S1.

## 3. Discussion

The present study shows that both DI TNC1 astroglia and astrocytes from hippocampal brain slices respond to GABA via the activation of GABA_A_ receptors, inducing a large increase in Cx43 hemichannel activity, and the Cx43 hemichannel-dependent release of glutamate and ATP. Experiments on DI TNC1 astrocytes suggest that GABA_B_ receptors and Panx1 channels may not have a measurable role in GABA-induced gliotransmission. This idea is supported by the fact that in hippocampal slices, Cx43 hemichannel activity, as well as glutamate and ATP gliotransmission, are all completely prevented by GABA_A_ antagonism and Cx43 hemichannel blockade, leaving very little room for a contribution from GABA_B_ receptors or panx1 channels, and by the fact that neither the GABA_B_ antagonist, nor the Panx1 blocker, affected GABA-induced gliotransmission.

Astrocytes have been reported to express both ionotropic GABA_A_ receptors [11,12] and metabotropic GABA_B_ receptors [8,13]. Activation of GABA_A_ receptors in astrocytes leads to Cl^-^ efflux from astrocytes, inducing depolarizing currents, triggering astrocytic Ca^2+^ signaling via the activation of voltage-sensitive Ca^2+^ channels [20,22,23,30,37,38]. GABA_B_ receptor activation also induces increases in intracellular [Ca^2+^] involving Gi/o proteins and Ca^2+^ release from intracellular stores [19,24] via IP_3_ signaling [17,19,24,39] followed by gliotransmitter release [19,28,40]. The GABA transporter (GAT)-mediated Na+ symport also increases [Ca^2+^]_i_ through the Na^+^/Ca^2+^ exchanger [41,42]. Finally, GAT-3 activation stimulates the astroglial release of ATP/adenosine, decreasing excitatory transmission via activation of presynaptic adenosine receptors [41] and increasing inhibitory transmission via activation of postsynaptic adenosine receptors [43]. The different pathways by which GABA can trigger an increase in [Ca^2+^]_i_ in astrocytes have been proposed to work synergistically [43].

Glutamate release has been shown to occur in astrocytes via several pathways besides Cx43 hemichannels, including reverse flow of glutamate transporters [44,45], volume-regulated anion channels (VRACs) [9,46,47,48,49], cysteine-glutamate Xc- antiporter [50], anion channel bestrophin 1(Best1) [51,52], P2X_7_ receptors [53] and vesicular exocytosis [54,55,56,57,58,59,60]. Our results show that glutamate release was entirely blunted by the Cx43 hemichannel blocker TAT-Cx43L2, suggesting that, in both DI TNC1 astrocytes and hippocampal slices, glutamate release in response to GABA is almost exclusively mediated by Cx43 hemichannels, ruling out an essential contribution from the other release mechanisms. There is, however, another possibility. Cx43 hemichannels are permeable to Ca^2+^, so another option is that the Ca^2+^ released from astroglial intracellular stores is insufficient to trigger glutamate release throughout the astroglial processes but may be sufficient to increase Cx43 hemichannel activity, which could, in turn, induce the localized Ca^2+^ influx required to activate other complementing release mechanisms in localized astroglial compartments. Hence, Cx43 hemichannel-dependent gliotransmitter release could begin when hemichannels open but could be complemented by other Ca^2+-^-dependent release mechanisms. This way, Cx43 hemichannel activity could be an early “upstream” step required for gliotransmission, which would explain why blocking Cx43 hemichannels completely blunts gliotransmission. Further research will be needed to assess this possibility.

Studies have shown that GABA induces the release of glutamate [25,26] and ATP/adenosine from astrocytes [25,27,28], with a net inhibitory effect. In the present study, the activation of GABA_A_ receptors led to increased Cx43 hemichannel activity and Cx43 hemichannel-dependent release of both glutamate and ATP via GABA_A_, which is congruent with several studies showing intracellular [Ca^2+^] increases in primary astrocytes in response to GABA_A_ activation in primary astroglial cultures [11,20,21,22]. However, a previous study found that astrocytes from adult cortical slices respond to GABA via GABA_B_ receptors [24]. In the present report, neither the increase in Cx43 hemichannel activity nor the release of glutamate and ATP in response to GABA was sensitive to the GABA_B_ receptor antagonist CGP55845. It may be argued that the concentration used was insufficient to attain effects, as a dose-response curve was not performed. The use of a dose-response curve was deemed unnecessary, as the blockade of GABA_A_ receptors by bicuculline completely abolished both Cx43 hemichannel activity increases and gliotransmission in response to GABA. Hence, the present results suggest that the main mechanism by which GABA induces an increase in Cx43 hemichannel activity and astroglial release of glutamate and ATP is via the activation of GABA_A_ receptors in both DI TNC1 astrocytes and hippocampal slices.

Previous studies have reported that astrocytes obtained from animals at post-natal P3 or P32–34 express primarily GABA_A_ receptors, and only 10% of them responded via GABA_B_ receptors. In contrast, those obtained from P11 and P15 showed 60% of astrocytes responding via GABA_B_ receptors [23]. Consequently, it is possible that the timing in which astrocytes were obtained could influence their GABA receptor expression. More research is needed to address how astrocytes decide to respond to GABA, either via GABA_A_ or GABA_B_ receptors, but based on current literature, our results support GABA_A_ as the main GABA receptor mediating the effects of GABA on astroglial gliotransmission.

One of the most interesting results of the present study is that GABA induces the astroglial release of both glutamate and ATP almost exclusively through Cx43 hemichannels, in both DI TNC1 and hippocampal slices. In previous studies performed in brain slices, it was reported that GABA caused the release of glutamate and ATP, but instead of inducing increased neuronal excitation, it produced an astrocyte-dependent decrease in neuronal excitability that was mediated by neuronal adenosine receptors [25,27,28]. Although it is unclear from those studies whether adenosine was released by the astrocytes or was produced by neurons from the ATP that was released from astrocytes, this result suggests that while astrocytes may release both glutamate and ATP, the synaptic effect resulting from their release may be dependent on different factors, which may include the neuronal expression of the enzyme that converts ATP into adenosine (ecto-5′-nucleotidase), the expression of adenosine receptors, or the possible release of adenosine from astrocytes, which was not measured in the present study.

This inhibitory effect found after the release of glutamate and ATP in response to GABA is particularly interesting because glutamate also induces the astroglial increase in [Ca^2+^]_i_ and the release of glutamate and ATP [29]. While the ATP released by astrocytes always induces a decrease in neuronal excitability [61,62], the glutamate released by astrocytes induces an increase in intracellular cAMP and IP_3_, Ca^2+^ influx, and an increase in excitability and plasticity in neurons [63]. In the case of glutamate, the net effect of glutamate-induced gliotransmission is excitatory [64]. It is possible that the amount of glutamate and ATP released by each astrocyte may be different in response to different neurotransmitters, or it is possible that a different set of gliotransmitters be released by different astrocytes. In the central amygdala, astrocytic ATP/adenosine has been reported to depress excitatory neurotransmission by activating presynaptic A1 receptors and increasing the transmission of inhibitory inputs through the activation of presynaptic A2A receptors [65]. However, studies performed in the basolateral amygdala have shown that astroglial Cx43 hemichannels regulate post-synaptic NMDAR receptor activity via released glutamate and D-serine but not ATP [36]. Hence, the gliotransmitters released, as well as the receptors and enzymes expressed by neighboring neurons, may determine the final neuronal response to gliotransmitters. A recent review noted that this paradoxical effect might be related to mechanisms of astroglial integration [66]. How the astroglial release of glutamate, ATP, and other gliotransmitters may induce excitation and inhibition is an interesting question that remains unanswered and warrants further research.

Many psychiatric disorders, including anxiety-related disorders and depression, have been associated with a dysfunction in GABAergic transmission [67,68], and both effective antidepressant treatments and ECT have been shown to augment brain GABA [69]. Previous studies have also reported increased astroglial Cx43 hemichannel activity, both in post-mortem samples from depressed patients [70] and in animal models for depression [71]. In the latter, we found that the chronic restraint stress commonly used to induce depression-like symptoms in rodents causes an increase in Cx43 hemichannel activity in astrocytes and a Cx43 hemichannel-dependent increase in extracellular glutamate and ATP in the ventral hippocampus [71]. Whether this increase in glutamate and ATP leads to synaptic excitation or inhibition is still unknown. However, astroglial dysfunction may contribute to psychiatric disorders like depression by affecting the excitatory/inhibitory balance in different brain regions. Astrocytes can affect this balance by changing the expression and availability of GABA and glutamate transporters. They may also release glutamate and ATP, which depending on the surrounding neurons, may induce synaptic excitation or inhibition [66]. Consequently, the contribution of Cx43 hemichannel-dependent astroglial gliotransmission to the pathogenesis of psychiatric disorders, like depression, is a theme that will keep researchers busy for the next years to come.

## 4. Materials and Methods

### 4.1. DI TNC1 Astroglial Cell Culture

The rat astrocyte cell line DI TNC1 (ATCC CRL-2005) was cultured at 37 °C, and 5% CO_2_ in Dulbecco’s Modified Eagle’s Medium (DMEM) supplemented with 5% fetal bovine serum (Life Technologies, Carlsbad, CA, USA) and 100 µM/mL streptomycin and ampicillin (1:1, 100 IU/mL). The culture medium was replaced every other day.

### 4.2. DAPI Uptake

To measure Cx43 hemichannel activity, snapshots of DAPI uptake in DITNC1 astrocytes were taken, as reported in previous studies [71]. In summary, once the cells reached 80% confluence, they were transferred to 96 well plates with a HEPES -buffered salt solution either with Ca^2+^ (140 mM NaCl, 4 mM KCl, 1 mM CaC1_2_, 1 mM MgC1_2_, 5 mM glucose, 10 nΜ HEPES) or divalent cations (Ca^2+^)-free (140 mM NaCl, 4 mM KC1, 5 mM glucose, 2 mM EGTA, 10 nM HEPES). The Ca^2+^-free solution was used to assess whether GABA or bicuculline can decrease Cx43 hemichannel opening, as hemichannels open when cells are incubated in a Ca^2+^-free medium [30]. The standard solution containing 1 mM Ca^2+^ was used to determine whether GABA or bicuculline can trigger the opening of Cx43 hemichannels, as in normal-physiological Ca^2+^ conditions, the Cx43 hemichannel opening probability is low [30]. The ligands were added to both solutions for 5 min, followed by 5 min incubation with 4′,6-diamidino-2-phenylindole dihydrochloride (DAPI, Sigma-Aldrich, St. Louis, MO, USA, 10 µM).

Cells were then washed to eliminate extracellular DAPI and examined in fluorescence microscopy using a FLoid^®^ Cell Imaging Station (Invitrogen, Waltham, MA, USA). Each well was photographed under white light and fluorescence (excitation, 340 nm; emission, 488 nm), using parameters preset under control conditions (no ligand). Each photograph was subdivided into four quadrants, and all cells in one quadrant were counted under white light (total cells) and fluorescence for the emission of DAPI (fluorescent nuclei). Only quadrants having at least 250 cells were included in the analysis. This way, the percentage of fluorescent cells with DAPI-labeled nuclei from the total number of cells present in the photograph was estimated for each ligand concentration. To determine the EC50 for GABA, the percentage of increase in the number of fluorescent cells was plotted from the uptake experiments for each concentration, and the concentration needed to attain a 50% effect was extrapolated. To determine whether the effects depended on intracellular Ca^2+^, cells were preincubated with cell-permeable Ca^2+^ chelator BAPTA-AM (Sigma-Aldrich, St. Louis, MO, USA) at 100 nM, before adding the ligand. To corroborate that the DAPI uptake depended specifically on Cx43 hemichannels, cells were preincubated with 10 nM of the selective Cx43 hemichannel blocker peptide TAT-Cx43L2 (YGRKKRRQRRRDGANVDMHLKQIEIKKFKYGIEEHGK; LifeTein, NJ, Somerset, USA;) or with 10 nM of its control mutant TAT-Cx43L2^H126K/I130N^ (Biomatik, Kitchener, ON, Canada), before the addition of the ligands.

### 4.3. Drugs Evaluated

The effect of GABA (Sigma-Aldrich) in DI TNC1 astrocytes was evaluated using six different concentrations of GABA, ranging from 1 nM to 100 µM. The experiment was performed in three wells and repeated 2 more times to obtain triplicates (N = 3, with technical triplicates). The 1 μM concentration was the lowest concentration with a significant effect. The GABA_A_ antagonist Bicuculline (Sigma-Aldrich) was evaluated at six concentrations from 1 nM to 100 μM for 5 min. The maximal concentration evaluated did not show an effect by itself (100 µM). Then, cells were pre-incubated with bicuculline at 100 µM and then incubated with GABA (1 μM, Sigma-Aldrich). The GABA_B_ antagonist CGP55845 (Tocris, Bristol, UK) was evaluated at 1 µM, the panx1 blocker peptide ^10^Panx1 (Genscript, Piscataway, NJ, USA) at 150 µM and the mutant TAT-Cx43L2^H126K/I130N^ (Biomatik, Kitchener, ON, Canada) was evaluated at 10 nM. All drugs were dissolved in PBS.

### 4.4. Measurement of Extracellular Glutamate and ATP

The cells were transferred to 12 well plates with 1,2 mL of a HEPES -buffered salt solution with Ca^2+^ (140 mM NaCl, 4 mM KCl, 1 mM CaC12, 1 mM MgC12, 5 mM glucose, 10 nΜ HEPES). GABA (1 µM) was added either alone, or after pretreatment with TAT-Cx43L2 (10 nM) or bicuculline (100 µM) for ten minutes. After five minutes of incubation with GABA, the medium without cells was transferred to 1 mL aliquots and frozen at −80 °C and then thawed before gliotransmitter analysis. Extracellular ATP was measured using a luciferin/luciferase bioluminescence assay kit (Sigma-Aldrich). Extracellular glutamate levels were determined using a colorimetric enzyme-linked assay (Sigma-Aldrich). In the presence of glutamate dehydrogenase (GDH) and β-nicotinamide adenine dinucleotide phosphate (NADP^+^), glutamate is oxidized to α-ketoglutarate, yielding NADPH, which can be determined fluorometrically for indirect quantification of glutamate concentration (excitation and emission wavelengths of 355 nm and 460 nm, respectively). For each assay, standard curves were constructed using standardized ATP and glutamate concentrations used to estimate the concentrations of ATP and glutamate in the samples, which are shown per 10^6^ cells for in vitro experiments and per mL for hippocampal slices (ex vivo).

### 4.5. Animals

All procedures involving animals were performed in accordance with NIH guidelines and with the approval of the bioethical committee of the Universidad Andrés Bello (Acta 003-2020). Sprague Dawley male rats (~60-day-old, ~250 g) were caged individually at 22 °C under a 12/12 h light/dark cycle and given food chow and water ad libitum.

### 4.6. Hippocampal Slice Preparation

Dye uptake “snapshot” assays were performed as reported previously [36,71], with minor modifications. In brief, naïve rats were anesthetized under isoflurane. They were decapitated and their brains were extracted and cut into coronal slices (400 µm) in an ice-cold slicing solution containing (in mM): sucrose (222); KCl (2.6); NaHCO_3_ (27); NaHPO_4_ (1.5); glucose (10); MgSO_4_ (7); CaCl_2_ (0.5) and ascorbate (0.1), bubbled with 95% O_2_/5% CO_2_, using a vibratome (Leica, VT1000GS; Wetzlar, Germany). Then, the slices were transferred to a holding chamber filled with artificial cerebral spinal fluid (ACSF), at room temperature (20–22 °C), containing (in mM): 125 NaCl, 2.5 KCl, 25 glucose, 25 NaHCO_3_, 1.25 NaH_2_PO_4_, 2 CaCl_2_, and 1 MgCl_2_, bubbled with 95% O_2_/5% CO_2_, pH 7.4, for a stabilization period of 60 min before dye uptake experiments.

### 4.7. Dye Uptake Measurements in Hippocampal Slices

For dye uptake and ex vivo ‘‘snapshot’’ experiments, the slices were incubated with 5 µM ethidium (Etd^+^) for 10 min in a chamber oxygenated by bubbling 95% O_2_/5% CO_2_ into ACSF. To ascertain that the dye uptake was mediated by Cx43 hemichannels, some of the brain slices were incubated with 10 nM TAT-Cx43L2. To assess the contribution of GABA_A_ receptors, some slices were preincubated with bicuculine (100 µM). Preincubation was performed for 10 min, and then GABA was added at 100 µM for 5 min, followed by Etd^+^ for 10 min. After the 10-min exposure to Etd^+^, the slices were washed three times with ACSF and were fixed at room temperature with 4% saccharose in 4% paraformaldehyde for 60 min, rinsed once for 5 min with 0.1 mM glycine in PBS, and then twice with PBS for 10 min with gentle agitation. Then the slices were sectioned using a cryostat to obtain ~ ten sections (40 µm each). The sections from the slices were incubated two times for 30 min each with a blocking solution (PBS, gelatin 0.2%, Triton-X 100 1%) at room temperature and then incubated overnight at 4 °C with a cell-specific antibody to identify astrocytes (anti-GFAP monoclonal antibody; Sigma-Aldrich). After the incubation with the antibody diluted 1:1000 in the blocking solution, the slices were washed for 10-min with the blocking solution, 3 times. The sections were then incubated for 2 h at room temperature with donkey anti-mouse Alexa Fluor 488 antibody (1:1.000, Abcam) and after 3 washes (10-min each), the slices were mounted in Fluoromount, cover-slipped, and examined in an epifluorescence microscope (ECLIPSE Ti-E, Nikon, Tokyo, Japan). Stacks of consecutive images were taken with a 60× objective at 1-µm intervals, acquired sequentially at two wavelengths (488 nm and 543 nm), and Z stack projections were reconstructed using ImageJ software (National Institute of Health, Bethesda, MD, USA). Dye uptake was calculated as: corrected total cell Etd fluorescence intensity (as arbitrary units [AU]) = integrated density − ([area of selected cell] × [mean fluorescence of background readings]). At least six cells per field were selected from at least three fields in each brain slice. The background fluorescence was set as the average value of intensity in three regions of interest (ROI) void of any fluorescence.

To measure fluorescence intensity, ROIs in GFAP positive cells (astrocytes) were set surrounding cell nuclei and the fluorescent intensity was measured using ImageJ for each cell, for all cells in each quadrant, from the same quadrants that were analyzed for counting DAPI labeled cells, from the GABA and control conditions (*n* = 3 with triplicates). Fluorescent intensities were plotted for all fluorescence-labeled cells, and the median number of cells and median fluorescence were compared for each condition.

### 4.8. Measurements of Extracellular Glutamate and ATP

The same protocol for hippocampal slices was used as above, but instead of adding Etd^+^, the slices were removed from the ACSF medium, which was transferred to 1 mL aliquots and frozen at −80 °C. For ATP and glutamate measurements, the aliquots were thawed and processed with the same procedure as explained above.

### 4.9. Statistics

All results were analyzed using the Prism software and were tested using the Kolmogorov-Smirnov test for normality and found to be normally distributed. Groups were compared either using one- or two-way ANOVA as required and a Tukey post-hoc test for multiple comparisons. Differences were considered significant when *p* ˂ 0.05 and shown as *p* ˂ 0.05 *, *p* ˂ 0.01 **, and *p* ˂ 0.001 ***.

## Data Availability

Not applicable.

## Supplementary Materials

The following supporting information can be downloaded at: https://www.mdpi.com/article/10.3390/ijms232113625/s1.
