# Supplementary material for: GABAergic Regulation of Astroglial Gliotransmission through Cx43 Hemichannels
Source: Int J Mol Sci. 2022 Nov 7;23(21):13625. doi: 10.3390/ijms232113625 (PMC9656947; doi:10.3390/ijms232113625)
Supplement: Supplementary file 1 [file ijms-23-13625-s001.zip › ijms-1785005-supplementary.pdf]

Supplementary Figure S1

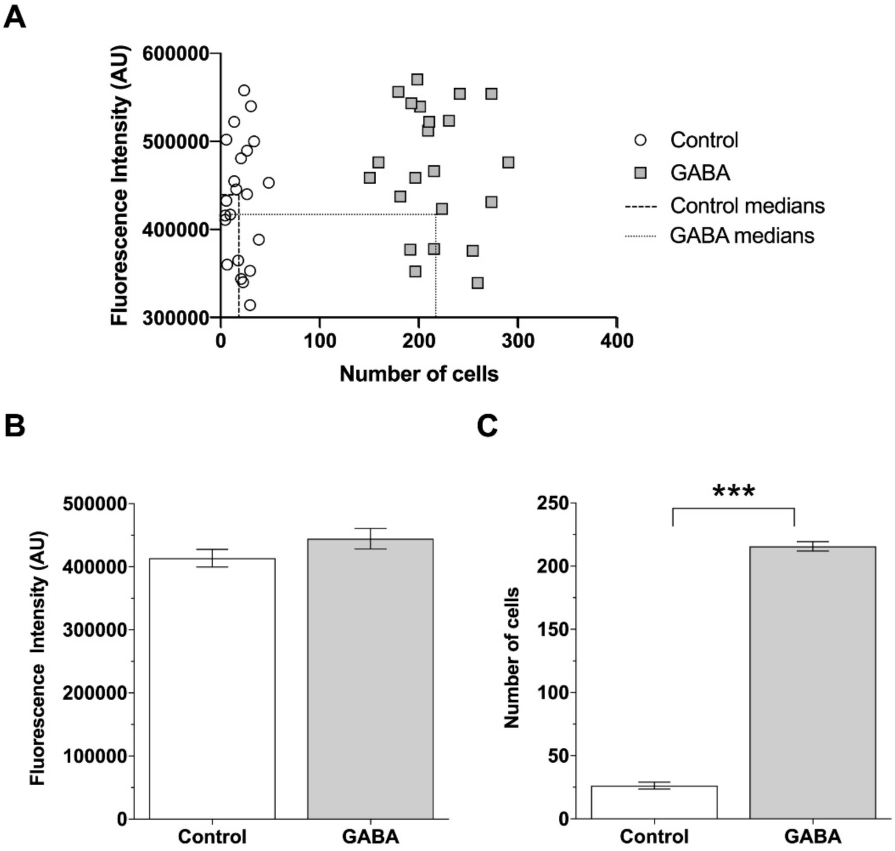

Figure S1. GABA induces an increase in the number of astrocytes with DAPI uptake but does not affect the median fluorescence intensity. (**A**) Plot of the number of DAPI labeled DI TNC1 astrocytes against their nuclear fluorescence intensity (control, white; GABA, black). Means are shown as dashed lines. (**B**) Comparison of fluorescence intensity means. (**C**) Comparison of the mean number of DAPI labeled cells. N=3 with technical triplicates. $p < 0.05$ *, $p < 0.01$ **, $p < 0.001$ ***.

Supplementary Table S1. Data and statistics

**DAPI uptake under calcium-free condition (DI TNC1 astrocytes)**			
**Treatment**	**Average (%) labeled/total cells**	**Comparison (ANOVA) ("-" separates compared groups)**	**Significance**
Control	91.06 ± 2.596	GABA-Control	NS
GABA	86.71 ± 2.596	GABA-GABA/TAT-L2	NS
GABA / TAT-L2	3.87 ± 2.596	GABA-GABA/BAPTA	NS
GABA / BAPTA-AM	3.03 ± 2.596	GABA-GABA/Bicuculline	NS
GABA / Bicuculline	86.79 ± 2.596	Control-TAT-L2	***
TAT-L2	5.69 ± 2.596	Control-BAPTA	***
BAPTA	1.11 ± 2.596	Control-Bicuculline	NS
Bicuculline	93.38 ± 2.596		

**DAPI uptake under normal calcium condition (DI TNC1 astrocytes)**			
**Treatment**	**Average (%) labeled/total cells**	**Comparison (ANOVA) ("-" separates compared groups)**	**Significance**
Control	4.57 ± 2.089	GABA-Control	***
GABA	93.15 ± 2.089	GABA-GABA/TAT-L2	***
GABA / TAT-L2	4.78 ± 2.089	GABA-GABA/BAPTA	***
GABA / BAPTA-AM	5.43 ± 2.089	GABA-GABA/Bicuculline	***
GABA / Bicuculline	9.53 ± 2.089	Control-TAT-L2	*
GABA / CGP	87.24 ± 4.384	Control-BAPTA	*
GABA / Panx1	75.33 ± 4.384	Control-Bicuculline	*
GABA / TAT-L2mut	82.50 ± 4.384	GABA-CGP	NS
TAT-L2	5.61 ± 2.089	GABA-^{10}Panx1	NS
BAPTA-AM	1.89 ± 2.089	GABA-TAT-L2mut	NS
Bicuculline	3.23 ± 2.089	Control-GABA	***
CGP	1.87 ± 4.384	Control-GABA/CGP	***
Panx1	2.40 ± 4.384	Control-GABA/^{10}Panx1	***
TAT-L2mut	2.15 ± 4.384	Control-GABA/TAT-L2Mut	***

DAPI fluorescence intensity under normal calcium condition (DI TNC1 astrocytes)			
**Treatment**	**Mean intensity (AU)**	**Comparison (t-test) ("-" separates compared groups)**	**Significance**
Control	413581	Control-GABA	NS
GABA	444448		

Glutamate release under normal calcium condition (DI TNC1 astrocytes)			
**Treatment**	**Average (nmol/10^6 cells)**	**Comparison (ANOVA) ("-" separates compared groups)**	**Significance**
Control	12.80 ± 2.951	GABA-Control	*
GABA	61.62 ± 2.951	GABA-GABA/TAT-L2	*
GABA / TAT-L2	14.72 ± 2.951	GABA-GABA/BAPTA	*
GABA / BAPTA-AM	13.49 ± 2.951	GABA-GABA/Bicuculline	*
GABA / Bicuculline	14.14 ± 2.951	Control-TAT-L2	NS
GABA / CGP	54.53 ± 1.758	Control-BAPTA	NS
GABA / Panx1	56.84 ± 1.758	Control-Bicuculline	*
GABA / TAT-L2mut	56.84 ± 1.758	GABA-CGP	NS
TAT-L2	11.02 ± 2.951	GABA-[10]Panx1	NS
BAPTA-AM	14.57 ± 2.951	GABA-TAT-L2mut	NS
Bicuculline	12.42 ± 2.951	Control-GABA	***
CGP	17.23 ± 1.758	Control-GABA/CGP	***
Panx1	18.71 ± 1.758	Control-GABA/[10]Panx1	***
TAT-L2mut	18.71 ± 1.758	Control-GABA/TAT-L2Mut	***

ATP release under normal calcium condition (DI TNC1 astrocytes)			
**Treatment**	**Average (nmol/10^6 cells)**	**Comparison (ANOVA) ("-" separates compared groups)**	**Significance**
Control	12.80 ± 2.392	GABA-Control	***
GABA	34.76 ± 2.392	GABA-GABA/TAT-L2	***
GABA / TAT-L2	10.18 ± 2.392	GABA-GABA/BAPTA	***
GABA / BAPTA-AM	10.73 ± 2.392	GABA-GABA/Bicuculline	***
GABA / Bicuculline	10.83 ± 2.392	Control-TAT-L2	NS
GABA / CGP	32.36 ± 1.656	Control-BAPTA	NS
GABA / Panx1	31.90 ± 1.656	Control-Bicuculline	NS
GABA / TAT-L2mut	32.49 ± 1.656	GABA-CGP	NS
TAT-L2	14.38 ± 2.392	GABA-[10]Panx1	NS
BAPTA-AM	14.37 ± 2.392	GABA-TAT-L2mut	NS
Bicuculline	13.81 ± 2.392	Control-GABA	***
CGP	15.38 ± 1.656	Control-GABA/CGP	***

Panx1	14.11 ± 1.656	Control-GABA/[10]Panx1	***
TAT-L2mut	13.06 ± 1.656	Control-GABA/TAT-L2Mut	***

**Etd uptake in hippocampal slices**			
**Treatment**	**Average (AU)**	**Comparison (ANOVA) (“-“ separates compared groups)**	**Significance**
Control	52.11 ± 10.11	GABA-Control	*
GABA	199.3 ± 10.11	GABA-GABA/TAT-L2	*
GABA / TAT-L2	56.75 ± 10.11	GABA-GABA/Bicuculline	*
GABA / Bicuculline	62.65 ± 10.11	Control-TAT-L2	*
TAT-L2	48.18 ± 10.11	Control-Bicuculline	*
Bicuculline	52.05 ± 10.11		

**Glutamate release in hippocampal slices**			
**Treatment**	**Average (nmol/mL)**	**Comparison (ANOVA) (“-“ separates compared groups)**	**Significance**
Control	33.87 ± 3.120	GABA-Control	*
GABA	144.0 ± 3.120	GABA-GABA/TAT-L2	**
GABA / TAT-L2	31.76 ± 3.120	GABA-GABA/Bicuculline	*
GABA / Bicuculline	34.05 ± 3.120	Control-TAT-L2	**
TAT-L2	31.14 ± 3.120	Control-Bicuculline	*
Bicuculline	33.28 ± 3.120		

**ATP release in hippocampal slices**			
**Treatment**	**Average (nmol/mL)**	**Comparison (ANOVA) (“-“ separates compared groups)**	**Significance**
Control	13.98 ± 32.66	GABA-Control	**
GABA	167.50 ± 32.66	GABA-GABA/TAT-L2	**
GABA / TAT-L2	14.73 ± 32.66	GABA-GABA/Bicuculline	**
GABA / Bicuculline	10.43 ± 32.66	Control-TAT-L2	NS
TAT-L2	16.75 ± 32.66	Control-Bicuculline	NS
Bicuculline	13.46 ± 2.089		

Differences were considered significant when $p < 0.05$ and shown as $p < 0.05$ *, $p < 0.01$ **, and $p < 0.001$ ***. NS, not significant.
